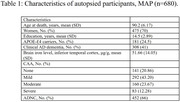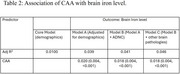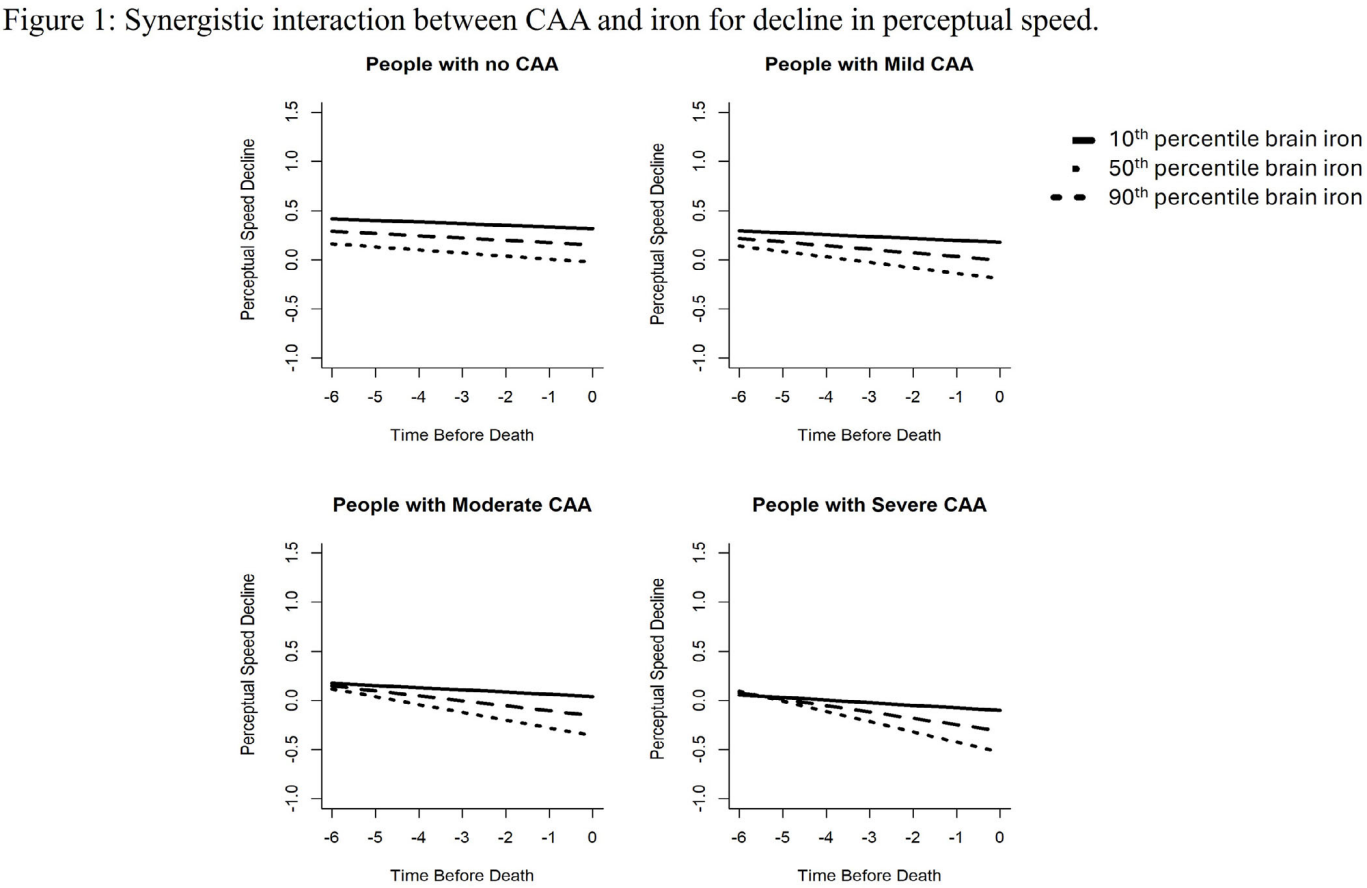# Role of brain iron on cerebral amyloid angiopathy and decline in cognition specifically perceptual speed in older people

**DOI:** 10.1002/alz70855_102215

**Published:** 2025-12-23

**Authors:** Sonal Agrawal, Sue E. Leurgans, Scott Ayton, Maude Wagner, Puja Agarwal, David A. A. Bennett, Ashley I. Bush, Julie A Schneider

**Affiliations:** ^1^ Rush Alzheimer's Disease Center, Rush University Medical Center, Chicago, IL, USA; ^2^ The Florey Institute of Neuroscience and Mental Health, The University of Melbourne, Australia, Melbourne, VIC, Australia; ^3^ Rush Alzheimer's Disease Center, Chicago, IL, USA

## Abstract

**Background:**

Cerebral amyloid angiopathy (CAA) is a common brain pathology associated with Alzheimer's Disease (AD) and cognitive decline. Iron is an essential micronutrient for brain health; elevated brain iron is associated with cognitive decline in our prior studies. This study aims to explore the association between CAA and brain iron and to examine whether the association between CAA and cognitive decline is modified by elevated iron levels. We hypothesized that persons with CAA would exhibit higher brain iron levels, and that higher iron levels would contribute to accelerated cognitive decline independently of AD neuropathologic changes (ADNC).

**Method:**

Data came from the Rush Memory and Aging Project (*N* = 680, mean age at death 90 ± 6.1 years, 70% women). Participants completed baseline and longitudinal cognitive assessments and underwent detailed neuropathologic evaluation for CAA, ADNC, and other brain pathologies. Severity of CAA (none, mild, moderate and severe) was assessed from the four neocortical regions using immunohistochemistry against Aβ antibody. Brain iron level in the inferior temporal cortex was assessed using Inductively Coupled Plasma Mass Spectrophotometry and a composite mean z‐score was generated. Linear regression and mixed‐effects models, adjusted for age‐at‐death, sex, education, ADNC, and other pathologies, were used for analysis.

**Result:**

The mean follow‐up before death was 6.7 ± 3.8 years. Over one‐third of participants (*N* = 243) had moderate‐to‐severe CAA, and mean brain iron was 52 (SD=14) μg/g. CAA was associated with higher iron levels (Est=0.018, SE=0.004, *p* = <0.001). When examining associations with cognitive change, both CAA and elevated iron level were independently associated with faster decline in global cognition and three cognitive domains (episodic, semantic, and perceptual speed) (all *p* <0.024). High iron was also associated with steeper decline in working memory (*p* = 0.001); CAA was associated with steeper decline in visuospatial orientation (*p* = 0.018). Furthermore, in participants with high iron levels, CAA was associated with steeper perceptual speed decline (Est=‐0.125, SE=0.048, *P=0.008*). No other interactions were found between CAA and iron for cognitive decline.

**Conclusion:**

Together, these findings demonstrate that the level of brain iron is associated with the clinical impact of CAA in older age.